# Mitigating climate disruption in time: A self-consistent approach for avoiding both near-term and long-term global warming

**DOI:** 10.1073/pnas.2123536119

**Published:** 2022-05-23

**Authors:** Gabrielle B. Dreyfus, Yangyang Xu, Drew T. Shindell, Durwood Zaelke, Veerabhadran Ramanathan

**Affiliations:** ^a^Institute of Governance & Sustainable Development, Washington, DC 20016;; ^b^Department of Physics, Georgetown University, Washington, DC 20057;; ^c^Department of Atmospheric Sciences, College of Geosciences, Texas A&M University, College Station, TX 77843;; ^d^Earth and Climate Sciences Division, Nicholas School of the Environment, Duke University, Durham, NC 27708;; ^e^Bren School of Environmental Science & Management, University of California, Santa Barbara, CA 93106;; ^f^Scripps Institution of Oceanography, University of California San Diego, La Jolla, CA 92037;; ^g^College of Agriculture and Life Sciences, Cornell University, Ithaca, NY 14853

**Keywords:** climate mitigation, short-lived climate pollutants, fossil fuel radiative forcing, near-term warming, non-CO_2_ climate effects

## Abstract

The ongoing and projected impacts from human-induced climate change highlight the need for mitigation approaches to limit warming in both the near term (<2050) and the long term (>2050). We clarify the role of non-CO_2_ greenhouse gases and aerosols in the context of near-term and long-term climate mitigation, as well as the net effect of decarbonization strategies targeting fossil fuel (FF) phaseout by 2050. Relying on Intergovernmental Panel on Climate Change radiative forcing, we show that the net historical (2019 to 1750) radiative forcing effect of CO_2_ and non-CO_2_ climate forcers emitted by FF sources plus the CO_2_ emitted by land-use changes is comparable to the net from non-CO_2_ climate forcers emitted by non-FF sources. We find that mitigation measures that target only decarbonization are essential for strong long-term cooling but can result in weak near-term warming (due to unmasking the cooling effect of coemitted aerosols) and lead to temperatures exceeding 2 °C before 2050. In contrast, pairing decarbonization with additional mitigation measures targeting short-lived climate pollutants and N_2_O, slows the rate of warming a decade or two earlier than decarbonization alone and avoids the 2 °C threshold altogether. These non-CO_2_ targeted measures when combined with decarbonization can provide net cooling by 2030 and reduce the rate of warming from 2030 to 2050 by about 50%, roughly half of which comes from methane, significantly larger than decarbonization alone over this time frame. Our analysis demonstrates the need for a comprehensive CO_2_ and targeted non-CO_2_ mitigation approach to address both the near-term and long-term impacts of climate disruption.

## Significance

This study clarifies the need for comprehensive CO_2_ and non-CO_2_ mitigation approaches to address both near-term and long-term warming. Non-CO_2_ greenhouse gases (GHGs) are responsible for nearly half of all climate forcing from GHG. However, the importance of non-CO_2_ pollutants, in particular short-lived climate pollutants, in climate mitigation has been underrepresented. When historical emissions are partitioned into fossil fuel (FF)- and non-FF-related sources, we find that nearly half of the positive forcing from FF and land-use change sources of CO_2_ emissions has been masked by coemission of cooling aerosols. Pairing decarbonization with mitigation measures targeting non-CO_2_ pollutants is essential for limiting not only the near-term (next 25 y) warming but also the 2100 warming below 2 °C.

Global warming is causing climate disruption today. At about 1.1 °C warming above preindustrial temperature (1), these impacts are being felt sooner and more intensely than previously projected (2). The frequency and intensity of climate and weather extremes have increased due to human-induced climate changes (1), and impacts such as displacements due to extremes are expected to grow with additional global warming (2).

We make a distinction between near-term warming and long-term warming: Near-term warming refers to the warming from now until 2050, while long-term refers to the period beyond 2050. This distinction omits the “mid-term (2041 to 2060)” recently introduced in the Intergovernmental Panel on Climate Change’s (IPCC) Sixth Assessment Report (AR6) (1). When the focus is on long-term, decarbonization to reach net-zero carbon dioxide emissions should be the foremost goal. However, a new set of issues has emerged because of the link between warming and extreme weather (3) and the risk of crossing uncertain tipping points that increase with additional warming (1, 4).

Every region is experiencing extreme weather impacts from climate change (2, 5). The number of potentially fatal humid heat events doubled between 1979 and 2017 (6), while heat-related mortality in people over 65 y increased 53.7% (7). Such fatal humid heat events are expected to become common in the tropics at global average temperatures above 1.5 °C (8, 9). Increases in humid heat also reduce labor productivity, with current losses of annual gross domestic product up to 6% in tropical countries (7) and nonlinear increases under warming (10). Actions that limit warming to close to 1.5 °C would “substantially reduce projected losses and damages related to climate change in human systems and ecosystems, compared to higher warming levels, but cannot eliminate them all (*very high confidence*)” (2).

The critical need to curb near-term warming and limit warming to well below 2 °C requires broadening the zero carbon dioxide emissions approach, which focuses on mitigating the long-term warming, with other approaches that can quickly reduce the near-term warming by including non-CO_2_ warming pollutants as an additional major focus of climate mitigation actions. The science of non-CO_2_ warming pollutants dates back to 1975 with the discovery of the supergreenhouse effect of chlorofluorocarbons (CFCs) (11) followed by the addition of methane (CH_4_) and nitrous oxide (N_2_O) in 1976 (12). A comprehensive review of non-CO_2_ warming agents by a United Nations–commissioned group in 1985 (13) concluded that non-CO_2_ greenhouse gases (GHGs) were contributing as much as CO_2_ to warming and projected that for the period between 1980 and 2030 non-CO_2_ gases were likely to continue contributing as much as CO_2_ to warming. These findings and projections have been confirmed by the most recent IPCC reports (14–17). We summarize these in the next section.

Independently, a series of studies that began in the 1970s concluded that fossil fuels (FFs), while contributing to global warming through CO_2_ emissions, were also leading to global dimming and resulting cooling by increasing atmospheric aerosol particles (18, 19). While the overall aerosol effect is strongly negative due to emissions of sulfates, nitrates, and some organics that primarily reflect sunlight, there are other aerosols such as black carbon (BC) and brown carbon that absorb sunlight and thus contribute to global warming. The findings of the three decades of studies have been confirmed by the most recent IPCC report, which concludes that as of 2019 the net radiative forcing from cooling aerosols is around –1.5 Wm^−2^ (excluding about +0.38 from the aerosol-radiation forcing from BC and its effect on surface albedo). The CO_2_ radiative forcing is 2.16 Wm^−2^ and radiative forcing due to non-CO_2_ GHGs and BC is 2.10 Wm^−2^ (15).

Despite the general recognition of the role of non-CO_2_ pollutants in climate mitigation, their contribution to warming as well as their potential for near-term cooling has been underappreciated in part due to inconsistencies between representation of climate forcing between IPCC Working Group I (WGI: Physical Scientific Basis), which includes all pollutants, and Working Group III (WGIII: Mitigation of Climate Change), which focuses on CO_2_ and the subset of GHGs covered under the Kyoto Protocol, hence excluding halogenated gases covered by the Montreal Protocol and both warming and cooling aerosols that are primarily coemitted with CO_2_ from FF usage. As we discuss in the next section, since FF combustion is the primary source of CO_2_ emissions and also the source of some non-CO_2_ pollutants, the extent to which decarbonization strategies to reduce FF emissions also reduce non-CO_2_ emissions is ambiguous in many mitigation studies due to study design, leading some to question the benefits of early and fast targeted action in reducing non-CO_2_ emissions (20).

The focus on CO_2_ underpins the concept of carbon budget, which has been used to construct decarbonization pathways to meet specified long-term warming levels (21). While it has long been known that the coincidental cancelling of non-CO_2_ warming and aerosol cooling was unlikely to persist due to differences in their sources and residence times (22), few carbon-budget-based studies have included the tight linkage between CO_2_ mitigation and reduction in cooling aerosol emissions until recently (23).

Many publications and reports by scientific agencies (24–32) highlighted the role of non-CO_2_ for rapid near-term climate mitigation, specifically short-lived climate pollutants (SLCPs)—methane (CH_4_), BC, hydrofluorocarbons (HFCs), and tropospheric ozone (O_3_)—but these have not captured the attention of global mitigation actions, which still focuses largely on CO_2_ emissions.

There are two primary objectives of this study: first, clarifying the role of non-CO_2_ GHGs (short-lived and long-lived) and aerosols (warming and cooling) in the context of the need for near-term and long-term climate mitigation, and second, clarifying the net effect of the FF phaseout in decarbonization, which involves both cooling due to cutting CO_2_ emissions and warming due to unmasking of cooling aerosols coemitted by FF use. Unless otherwise stated, we rely on forcing values in the IPCC WGI reports published in 2021 and 2013.

## Contributions to Radiative Forcing: CO_2_ vs. Non-CO_2_ GHGs (Excluding Aerosols)

**Fig. 1.**
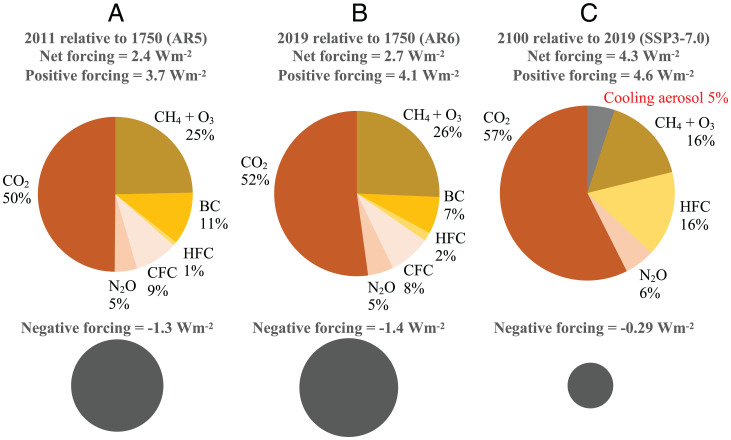
Positive radiative forcing from long-lived GHGs (orange), short-lived GHGs, GHG precursors, and BC (aerosol–radiation interaction and snow albedo effects only) (yellow) and negative forcing from individual aerosol direct effects (aerosol–radiation interaction) and the total aerosol indirect effects (aerosol–cloud interaction) (separate gray pie) in (*A*) 2011 relative to 1750, from AR5 (14) and (*B*) 2019 relative to 1750, from AR6 (15). (*C*) The forcing at 2100 relative to 2019, under SSP3-7.0 emissions (49). Note the negative forcing due to assumed BC and CFC reduction and the positive forcing due to decline of cooling aerosols. Area of each pie chart is scaled to positive or negative forcing. See *SI Appendix*, Fig. S5 for bar chart version and *SI Appendix*, Table S6*A* for data.

Previous reports of IPCC WGI have consistently found that CO_2_ and non-CO_2_ GHG and GHG precursor emissions contribute close to equal shares (52 to 57% for CO_2_ and 43 to 48% for non-CO_2_ GHG) to climate forcing in radiative forcing terms when excluding aerosols (*SI Appendix*, Table S1). These results are reproduced in Fig. 1 *A* and *B*. In contrast, IPCC WGIII states in the Fifth Assessment Report (AR5) that “CO_2_ emissions from fossil fuel combustion and industrial processes contributed about 78% of the total GHG emission increase from 1970 to 2010, with a similar percentage contribution for the period 2000–2010…. Annually, since 1970, about 25% of anthropogenic GHG emissions have been in the form of non-CO_2_ gases” (33). A similar statement was made by WGIII in the Fourth Assessment Report (AR4). However, these statements are inconsistent with WGI science and contribute to confusion for several reasons:

• First, GHG emissions considered by WGIII only include CO_2_ (from FF use and forestry and other land use, [FOLU]), CH_4_, N_2_O, and HFCs and omit nonmethane tropospheric ozone precursors, CFCs, hydrochlorofluorocarbons (HCFCs), and other ozone-depleting substances covered by the Montreal Protocol (*SI Appendix*, Fig. S1). Taking into account these omitted non-CO_2_ climate forcers using the EDGARv5.0 emissions database (34) for CO (as a proxy for nonmethane O_3_ precursors) and National Oceanic and Atmospheric Administration and AGAGE (35) network data for CFC/HCFC/halon emissions, the average non-CO_2_ GHGs and GHG precursors share over 1970 to 2010 is 39% (instead of the 25% quoted in WGIII reports) using the 100-y global warming potential (GWP_100_) metric and 59% using GWP_20_.
• Second, presenting the increase in emissions between two years (1970 and 2010) provides limited if not misleading insights into the actual forcing and climate impacts. We offer two examples, all of which adopt IPCC WGI estimates. 1) For the years 1993, 1998, 2005, 2011, and 2019, the percentage of CO_2_ forcing (from all sources) compared with the total GHGs forcing ranges from 52 to 57% (*SI Appendix*, Table S1). The non-CO_2_ GHGs contribute the balance of 43 to 48% (*SI Appendix*, Tables S1 and S2). 2) The contribution of the CO_2_ forcing from just FFs to the total GHGs forcing is 38% for 2011 and 43% for 2019. The basic inference is that the WGIII finding of “CO_2_ emissions from fossil fuel combustion and industrial processes contributed about 78% of the total GHG emission increase from 1970 to 2010” cannot be used to infer the contribution of CO_2_ or FFs to either the radiative forcing or the resulting climate changes.

In short, the conclusion by WGIII that CO_2_ from FF combustion contributed 78% of the total GHG emissions increase from 1970 to 2010 significantly underrepresents the nearly equal contribution of non-FFs as well as that of non-CO_2_ GHGs to the total radiative forcing, which are described in the next two sections. Revisiting this historical accounting puts into perspective the role of non-CO_2_ emissions in the current global warming and serves as a reminder of the need to consider all sources of climate forcing when assessing mitigation strategies.

This comparison of WGI and WGIII approaches also further underscores the importance of separately accounting for short- and long-lived pollutant emissions, as discussed by Daniel et al. (36) and recently called for by Allen et al. (37). Reporting these pollutants separately allows for consideration not only of potential effects of mitigation measures by source and implications for coemissions but also an assessment of temperature impact on multiple time horizons of interest (1). With 1.5 °C expected to be crossed in the early 2030s (1, 38), Abernethy and Jackson (39) have advocated for choosing time horizons for GHG aggregation metrics consistent with temperature goals, specifically supporting the use of GWP_20_ over the GWP_100_. A similar argument can be made in the context of the urgency to slow warming in the near term (2). In addition, common usage of aggregation metrics (e.g., GWP, GWP*, and global temperature potential) excludes very short-lived climate pollutants that are not well-mixed, such as aerosols and GHG precursors, but that can have significant implications for future warming (40, 41).

## Contributions to Radiative Forcing: FFs vs. Non-FFs (Including Aerosols)

**Fig. 2.**
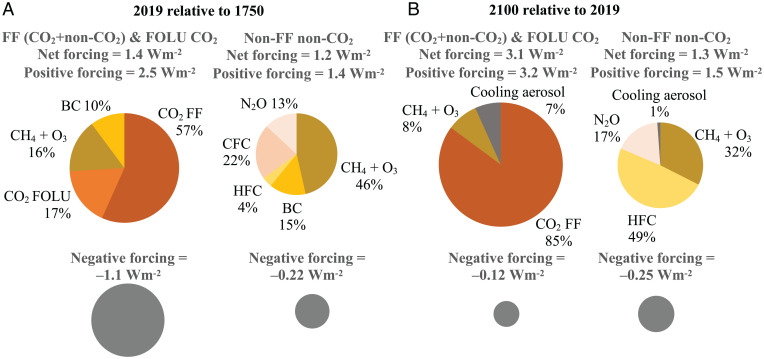
(*A*) Contributions to 2019 radiative forcing from emissions by FF (CO_2_+non-CO_2_) sources and CO_2_ from land-use changes (Forestry and Other Land Use, FOLU CO_2_) compared with emissions from non-FF non-CO_2_ sources based on ref. 42 and coemission factors from ref. 43 from this study, with similar results using radiative forcing values from AR6 WGI (*SI Appendix*, Table S3). (*B*) Contribution to the 2100 radiative forcing (relative to 2019) based on future emissions in SSP3-7.0 (49) partitioned by source using coemission factors from ref. 43. Area of each pie chart is scaled to positive or negative forcing. Data in *SI Appendix*, Table S6*B*.

Here we clarify the historical contributions to present-day radiative forcing from FF and non-FF sources. Many heat-trapping gases and particles originate from both FF and non-FF sources, while others such as N_2_O and halocarbons are primarily associated with non-FF sources. First, we calculate the relative share of emissions from FF and non-FF sources for GHGs alone, summing historical emissions pollutant by pollutant between 1850 and 2015 for each GHG based on source (42) and for future (after 2015) emissions using the FF coemission factors from Shindell and Smith (43) as described in *SI Appendix*. These shares are then applied to the total present-day radiative forcing in 2011 as in IPCC AR5 WGI (14) and 2019 as in IPCC AR6 WGI (15). Fig. 2 and *SI Appendix*, Table S2 show that for historical forcing (1750 to 2019) GHG from FF sources contributes about 53% of the total current GHG forcing, approximately the same as GHG forcing due to non-FF sources. However, if GHG emissions were to cease, residual forcing from long-lived GHG, predominantly FF CO_2_, would dominate as shorter-lived pollutants would be rapidly removed.

Next, we consider warming and cooling aerosols. For forcing estimates related to aerosols, we distinguish effective radiative forcing (ERF) due to aerosol-radiation interaction (ERF*_ari_*) for individual species from aerosol–cloud interaction (ERF*_aci_*) considered separately as a lump-sum “indirect” forcing term associated with total aerosol emissions (*SI Appendix*). Previous studies have shown that the coemission of aerosols from FF combustion can result in warming or cooling with distinct temporal and spatial patterns (27, 44). Many studies have identified the importance of cooling aerosols—primarily sulfates (with SO_2_ as the precursor), nitrates (NO, NO_2_, and NH_3_), and organic carbon—in masking GHG warming (1, 14). Fig. 1 shows the relative contributions of warming GHG, GHG precursors, and BC in comparison to the cooling from cooling aerosols relying on radiative forcing from historical emissions in recent IPCC reports, and how the relative contributions evolve in a reference scenario (SSP3-7.0) in 2100 relative to 2019.

The net forcings for all CO_2_ and non-CO_2_ FF (Fig. 2*A*) and non-FF non-CO_2_ (Fig. 2*B*) sources are based on Hoesly et al. (42) for the period through 2015. For 2016 to 2019, we use the Shared Socioeconomic Pathways (SSP) scenario and adopt Shindell and Smith’s (43) values for the coemission factors. We obtain similar results using radiative forcing values from AR6 WGI (*SI Appendix*, Table S3). For the radiative forcing from CO_2_ emitted by FF as well as non-FF sources and non-CO_2_ emitted by just FF, nearly half of the positive forcing (2.5 Wm^−2^) in 2019 is masked by negative forcing of cooling aerosols (–1.1 Wm^−2^), resulting in a net positive forcing of 1.4 Wm^−2^. The forcing of cooling aerosols from non-FF non-CO_2_ sources is only –0.2 Wm^−2^ compared to a positive forcing of 1.4 Wm^−2^. Thus, the net forcing from non-FF non-CO_2_ sources is 1.2 Wm^−2^ in 2019, or 45% of total net forcing when aerosols are included. The contribution to the net forcing from FFs (CO_2_ and other GHGs) is 39% when aerosols are included and from non-FF sources is 61%.

The picture depicted above changes in the projection through 2100 under the limited climate policy SSP3-7.0 scenario. By 2100, around 70% of net forcing relative to 2019 is due to FF and other CO_2_ emissions, emphasizing the importance of adopting decarbonization together with strategies targeting non-CO_2_ to address near-term and long-term warming.

## Contributions to Warming: CO_2_ vs. Non-CO_2_ and FFs vs. Non-FFs

The tendency to group CO_2_ and non-CO_2_ together irrespective of emission sources has contributed to a frequent misperception that CO_2_, which comes predominantly from FF burning, is the only important contributor to observed warming. This misperception is understandable: Our model shows that out of the 1.01 °C warming simulated for 2015, CO_2_ has contributed 0.98 °C (*SI Appendix*, Table S4). Thus, one can indeed claim that to the first order the observed global warming of ∼1 °C is primarily due to CO_2_. However, a closer look reveals that the magnitude of warming by non-CO_2_ GHGs coincidentally cancels the cooling by all (FF & non-FF sources) aerosols (45–47) (*SI Appendix*, Fig. S2). Indeed, our model shows that the combined cooling effects of aerosols including the indirect effects via enhancing cloud albedo (–1.15 °C) has masked an amount of warming that is almost equal to the total non-CO_2_ warming of 1.17 °C. This leads to a facile but false assumption that most non-CO_2_ forcings have canceled one another and will continue to do so in the future and obscures the significance of the residence time of the pollutants for both short- and long-term climate mitigation.

Uncovering the flaw in this reasoning requires correctly attributing the masking from cooling aerosols. Ignoring sources and aerosols, CO_2_ would appear to contribute about 55% of GHG warming (*SI Appendix*, Table S4). Considering only FF sources, *SI Appendix*, Table S4 shows that the warming from FF emissions (GHGs and BC) of 1.07 °C in 2015 is mostly masked by cooling of 0.88 °C from cooling aerosols that are coemitted with FF emissions. In contrast, while the warming from non-FF emissions (GHGs and BC) is equivalent in magnitude at 1.08 °C, only 0.26 °C is masked by coemitted cooling aerosols. This analysis reveals that about 80% of warming realized in 2015 is attributable to non-FF sources due to masking by cooling aerosols coemitted from FF sources. As these aerosols fall out of the atmosphere, the future net warming contribution from FF sources under SSP3-7.0 begins to dominate by the 2060s due to the longer residence time of CO_2_.

Accurately attributing past warming is key to mitigation actions going forward. As decarbonization measures reduce FF use they also reduce the coemitted cooling aerosols (primarily sulfates) and unmask the warming from accumulated GHGs in the atmosphere. In the following section we describe the implications of such unmasking for near- and long-term mitigation potential of decarbonization and clarify the essential role of strategies targeting non-CO_2_ pollutants in limiting warming through 2050.

## Mitigation Strategies in Time: Decarbonization and Targeted Mitigation

Reducing CO_2_ emissions by shifting from FF to low-carbon energy sources is underway and needs to accelerate to achieve net-zero CO_2_ emissions by midcentury or sooner consistent with the Paris temperature target (48). While getting to net-zero CO_2_ emissions is critical and essential for stabilizing long-term warming, it also reduces coemitted cooling aerosols and causes weak near-term warming, which can be offset by reductions in non-FF pollutants (43). Few studies, however, have specifically quantified the contribution of measures targeting non-CO_2_ independent from FF usage, such as the 16 measures in the 2011 UNEP/WMO Assessment (31).

Our analysis disentangles CO_2_, SLCPs, and cooling aerosols by asking the following question: Under an aggressive climate mitigation scenario (such as the marker version of SSP1-1.9), what is the avoided warming due to decarbonization alone (i.e., reduction in FF usage) and when paired with non-decarbonization-related mitigation targeting non-CO_2_ pollutants? We answer this question by explicitly accounting for the associated reductions in coemitted pollutants including cooling aerosols from each mitigation approach. As described in *SI Appendix*, we use SSP scenarios (49) and apply coemissions factors to partition emissions of individual pollutants into FF-related and non-FF-related (43). We consider three cases (Table 1): As a reference case we adopt the limited climate policy high-emission scenario SSP3-7.0, a middle case with only decarbonization-driven emissions reductions, and a “decarb+targeted” case including mitigation measures that go beyond decarbonization to target SLCPs and other non-CO_2_ pollutants (based on SSP1-1.9). We construct the “decarb-only” case by partitioning the reduction in emissions in the “decarb+targeted” case relative to the baseline case into decarbonization-driven and other targeted measures. Our approach differs from ref. 43 in that we use the SSP3-7.0 scenario to quantify the nondecarbonization mitigation potential from methane and BC. This includes mitigation measures targeting the ∼10% of methane emissions from abandoned coal mines and wells due to fugitive emissions that are not directly affected by decarbonization-driven reductions in FF use (*SI Appendix*).

**Table 1.** Simulated warming rates and other key metrics under reference, decarbonization only, and decarb+targeted scenarios

Scenario	Warming rate, °C/decade (2020–2040)	Year when warming rate drops below 0.25 °C/decade	Year of peak warming rate	Year when crossing 1.5 °C warming	Year when crossing 2 °C warming	Warming in 2030 relative to 1850–1900, °C	Warming in 2050 relative to 1850–1900, °C
Reference: Limited climate policy, high emission (SSP3-7.0)	0.36 (0.34–0.38)	—	—	2031–2033	2045–2046	1.5 (1.4–1.5)	2.2
Decarbonization-driven: Scenario using decreasing FF primary energy as in SSP1-1.9 and associated emission factors to calculate decline in FF-related emissions compared to reference	0.37 (0.35–0.39)	2049–2052	2030	2030–2032	2045–2046	1.5 (1.4–1.5)	2.1
Decarbonization and Targeted measures: Aggressive climate policy, low emission (based on SSP1-1.9)	0.31 (0.29–0.32)	2035–2037	2023	2030–2033	—*	1.5 (1.4–1.5)	1.85 (1.8–1.9)

The range of years reflects the uncertainty in present-day forcings of BC and cooling aerosols.

*Peak temperature of 1.9 °C in 2060s before declining to 1.7 °C in 2100.

All emission pathways including total and individual forcing were converted to temperature trajectories using the energy balance climate model RXM (*SI Appendix*), which has been validated in our earlier studies with climate models used in IPCC assessments (27, 30, 50, 51) and observed warming trends for the 20th century (*SI Appendix*, Fig. S3). Both the equilibrium and the transient climate sensitivity of the RXM model used in our study is within a few percent of the central values recommended in AR6. Our results for the avoided warming in the “decarb+targeted” case (*SI Appendix*, Table S5) are consistent with the results for methane, ozone precursor, and HFC abatement reported in AR6 WGI (52), which also used SSP3-7.0 as a reference case and SSP1-1.9 as the mitigation case, but do not account for source partitioning. With RXM we find avoided warming of 0.3 °C by 2040 from SLCP mitigation compared to 0.1 to 0.4 °C in AR6. The impact of SLCP reductions in 2100 is 0.5 to 1.3 °C in AR6, compared to 1.7 °C in our scenarios, which likely reflects the more stringent HFC and N_2_O reductions in our adapted mitigation scenario. Our methane mitigation benefit of ∼0.2 °C by 2050 is smaller than the ∼0.3 °C in a recent assessment based on similar abatement (38), suggesting that the sensitivity of RXM to methane is lower than that in the three-dimensions composition-climate models (but well within uncertainties) (*SI Appendix*).

**Fig. 3.**
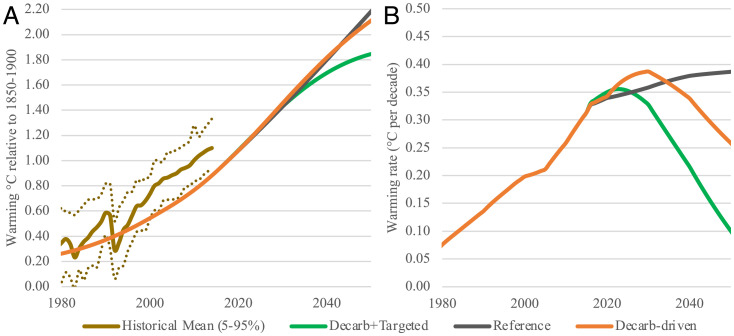
(*A*) Historical and future temperature projections through 2050 calculated using the RXM energy balance model based on emissions scenarios from the SSP database (49) for reference scenario (SSP3-7.0), decarbonization-driven mitigation scenario (this study), and an “decarb+targeted” scenario including aggressive decarbonization and targeted SLCP mitigation (adapted from SSP1-1.9). Historical curve (past simulated warming) is from figure SPM8.a (47, 64). (*B*) Rate of warming (degrees Celsius per decade) in the reference SSP3, decarbonization only, and “decarb+targeted” mitigation cases.

Aggressive decarbonization to achieve net-zero CO_2_ emissions in the 2050s (as in the decarb-only scenario) results in weakly accelerated net warming compared to the reference case, with a positive warming up to 0.03 °C in the mid-2030s and no net avoided warming until the mid-2040s due to the reduction in coemitted cooling aerosols (Fig. 3*A*). By 2050, decarbonization measures result in very limited net avoided warming (0.07 °C), consistent with Shindell and Smith (43), but rise to a likely detectable 0.25 °C by 2060 and a major benefit of 1.4 °C by 2100 (*SI Appendix*, Table S5).

In contrast, pairing decarbonization with mitigation measures targeting CH_4_, BC, HFC, and N_2_O (not an SLCP due to its longer lifetime) independent from decarbonization are essential to slowing the rate of warming by the 2030s to under 0.3 °C per decade (Table 1 and Fig. 3*B*), similar to the 0.2 °C to 0.25 °C per decade warming prior to 2020 (38, 53). Recent studies suggest that rate of warming rather than level of warming controls likelihood of record-shattering extreme weather events (54, 55).

By 2050, the net avoided warming from the targeted non-CO_2_ measures is 0.26 °C, almost four times larger than the net benefit of decarbonization alone (0.07 °C) (*SI Appendix*, Table S5). These results are calculated using an average BC forcing at present of 0.33 Wm^−2^ relative to preindustrial (direct and snow albedo; *SI Appendix*), which is consistent with the AR6 range (0.30 ± 0.2 Wm^−2^ for ERF*_ari_* and 0.38 Wm^−2^ including snow albedo effects) (56). Combining all targeted non-CO_2_ measures results in a net avoided warming in 2060 of 0.43 °C. Pairing decarbonization measures with targeted measures can achieve 0.25 °C in total avoided warming, a level that is likely to be detected (57) over a decade earlier (∼2047) than decarbonization alone (2060; *SI Appendix*, Table S5). The avoided warming due to decarbonization begins to exceed that due to the targeted measures only after 2080 (*SI Appendix*, Fig. S4).

Only about 30% of the avoided warming from CH_4_ over the period 2020 to 2040 is related to decarbonization measures (*SI Appendix*, Table S5). The larger portion of CH_4_ reduction due to targeted measures may be due to a slower rate of reduction in natural gas usage in the marker SSP1-1.9 scenario (60% down in 2050 relative to 2015) compared with decrease in coal combustion (more than 90% down). Consistently, about two-thirds of non-CH_4_-induced ozone mitigation is also due to non-CO_2_ targeted measures rather than a direct consequence of decarbonization. These results are also consistent with UNEP/WMO (31), which found that measures to reduce methane and BC emissions cut warming in 2030 by half compared with a reference case and that aggressive CO_2_ reductions, in themselves, did little to mitigate warming in the first 20 to 30 y, in part due to unmasking of coemitted cooling aerosol.

Fig. 3*A* shows that combining targeted mitigation strategies with decarbonization keeps warming below 2.0 °C, while decarbonization alone breaches 2.0 °C in 2045 in our scenario. Moreover, decarbonization alone increases the warming rate in the near term (Table 1). Notably, the warming rate in the decarbonization scenario would not drop below the current rate of warming until the 2040s (Fig. 3*B*). Pairing decarbonization with measures targeting SLCP slows the rate of warming a decade or two earlier than decarbonization alone.

## Consideration of Uncertainties

The largest uncertainties in our analysis relate to the mitigation pathways chosen, both the reference limited climate policy scenario and the low-emission mitigation scenarios. While current CO_2_ emissions commitments track closer to SSP2-4.5, the key insight of our study is not about additionality in terms of new policy measures. Rather, our study seeks to distinguish between mitigation policy focused on FF decarbonization alone versus decarbonization plus targeted measures. For this reason, we selected as a reference the high-emission scenario SSP3-7.0 and as a low-emission scenario SSP1-1.9, which are the same end-member scenarios as assessed in AR6 WGI (52).

The second major source of uncertainty is the nearly threefold uncertainty in climate sensitivity. All of the projected warming numbers presented here should be interpreted as median value with 50% probability. A third source of uncertainty relates to our use of constant FF coemission factors in constructing the decarbonization-driven scenario. Since this partitioning approach is most valid in the near term, we focus our analysis on the period through 2050. A fourth source of uncertainty relates to our limited understanding of the role of aerosols in climate forcing and feedbacks in future projections due to the following aspects: 1) the assumption of mixing of various aerosol species, especially the potential enhancement of BC forcing when accounting for the mixing with other reflective aerosols (58), 2) the future changes of background cloud field due to the slow feedback process to GHG warming (59, 60), and 3) the future changes of background aerosols from natural sources such as dust and sea salt due to climatic changes affecting the emission processes related to soil condition and wind stress over ocean surface and related cloud impacts (e.g., ref. 61).

## Conclusions

This study clarifies as well as establishes the need for a comprehensive and inclusive CO_2_ and non-CO_2_ mitigation approach with distinct decarbonization and SLCP targets to address both the near-term and long-term impacts of climate disruption. A review of IPCC reports leads to the inference that non-CO_2_ GHGs are responsible for nearly half of all current climate forcing from GHGs. When accounting for aerosols and coemissions by source, the inference from our analyses is that about 80% of the realized warming as of 2015 is attributable to non-FF sources due to FF GHG emissions being masked by coemission of short-lived cooling aerosols. However, the importance of non-CO_2_ pollutants, in particular SLCPs, and their role in climate mitigation has been underappreciated due to misperception arising from inconsistencies between IPCC WGI and WGIII reports. The tendency to attribute impact to pollutants rather than sources and to group all non-CO_2_ together regardless of emissions sources has further entrenched this misperception due to coincidental cancelling of warming and cooling pollutants and the false impression that they will continue to cancel out in the future. When historical emissions are partitioned into FF- and non-FF-related sources, we find that nearly half of the forcing from FF and other CO_2_ emissions has been masked by coemission of cooling aerosols. As a result, close to half of net radiative forcing, as of now, is attributable to non-FF sources of methane, F-gases, BC, and N_2_O. However, this is likely to change in the future as decarbonization policies reduce FF emissions of both warming GHGs and cooling aerosol.

By 2100, absent climate policy, FF will be the largest source (about 70%, mostly due to CO_2_) for global warming and resulting impacts on planet and society. Even in the shorter term, FF emissions are the largest source of air pollution particles and ozone, which contribute to premature mortality of over 8 million people per year (45, 62). Tropospheric ozone also leads to crop losses of 100 million tons or more (63). As we have repeatedly emphasized in this study, achieving net-zero carbon dioxide emissions by 2050 is essential to limit global warming below 2 °C beyond 2050.

Pairing decarbonization with targeted SLCP mitigation measures is essential to simultaneously limit both near-term warming and long-term warming below 2 °C and thus reduce risks from crossing tipping points. Importantly, these two strategies are complementary and not interchangeable. Absent deep cuts in non-CO_2_ emissions, CO_2_ abatement alone is unable to keep warming below even the 2 °C threshold by 2050. Decarbonization measures alone achieve about a third of potential avoided warming from methane mitigation by 2050, less than half of SLCP mitigation potential, and none of the reductions from measures targeting N_2_O. Nor can cutting methane emissions this decade replace the need for net-zero carbon dioxide by 2050 to stabilize the climate this century. Similarly, deeper CO_2_ reductions this decade do not replace the need for methane and other SLCP reductions to slow warming in the near term. Aggregation metrics such as GWP and GWP* are designed in terms of warming impacts over multiple decades and are seldom used in ways that account for the important differences between strategies that can reduce warming in the near term.

Adopting a comprehensive mitigation approach that pairs rapid decarbonization with “strong, rapid and sustained reductions in CH_4_ emissions” (1) as recommended in the Global Methane Assessment (32) and additional targeted SLCP mitigation responds to the call from WGII for urgent action to slow warming in the near term (2). For example, over 100 countries joined the Global Methane Pledge in November 2021, committing to a collective goal of reducing global anthropogenic methane emissions by at least 30% below 2020 levels by 2030. If achieved, this target, which is consistent with the reduction in the “decarb+targeted” scenario analyzed here, would avoid 0.2 °C by 2050 (*SI Appendix*, Table S5).

## Supplementary Material

Supplementary File

## Data Availability

All study data are included in the article and/or *SI Appendix*.
