# Supplementary material for: Mitigating climate disruption in time: A self-consistent approach for avoiding both near-term and long-term global warming
Source: Proc Natl Acad Sci U S A. 2022 May 23;119(22):e2123536119. doi: 10.1073/pnas.2123536119 (PMC9295773; doi:10.1073/pnas.2123536119)
Supplement: Supplementary File [file pnas.2123536119.sapp.pdf]

Supplementary Information for

Mitigating Climate Disruption in Time: a self-consistent approach for avoiding both near-term and long-term global warming

Gabrielle B. Dreyfus[1,2], Yangyang Xu[3**], Drew T. Shindell[4], Durwood Zaelke[1,5], and Veerabhadran Ramanathan[6,7*]

[1] Institute of Governance & Sustainable Development, Washington, DC 20016;

[2] Department of Physics, Georgetown University, Washington, DC 20057;

[3] Department of Atmospheric Sciences, College of Geosciences, Texas A&M University, College Station, TX 77843;

[4] Earth and Climate Sciences Division, Nicholas School of the Environment, Duke University, Durham, NC 27708;

[5] Bren School of Environmental Science & Management, University of California Santa Barbara, Santa Barbara, CA 93106;

[6] Scripps Institution of Oceanography, University of California San Diego, La Jolla, CA 92037;

[7] College of Agriculture and Life Sciences, Cornell University, Ithaca, NY 14853

*Corresponding author: Veerabhadran Ramanathan, Scripps Institution of Oceanography, University of California, San Diego, 9500 Gilman Drive, MC 0221, La Jolla, CA 92093-0221; (858) 534-0219

Email: vramanathan@ucsd.edu

** Second corresponding author: Yangyang Xu, Department of Atmospheric Sciences, Texas A&M University, 1204 Eller O&M, 3150 TAMU, College Station, TX 77843-3150; (979) 845-8076 Email: yangyang.xu@tamu.edu

1

Materials and Methods

This paper conducts a quantitative modelling exercise for a combination of more than a dozen individual species including CO_2, N_2O, ozone-depleting substances such as CFCs and HCFCs, various cooling aerosols such as sulfate, NO_x, organic carbon, and the well-known group of SLCPs including black carbon, ozone, methane, and HFCs. The modelling exercise produces the relative contribution of those species to historical (1850–2015) and future (2016–2100) warming.

The IPCC AR6 WGIII report was published the same week as we received galley proofs for our paper, so we are not commenting further beyond noting that an initial review did not change our findings.

Historical decomposition into fossil-fuel related and non-fossil fuel related emissions are based on Hoesly et al. (1). For sulfate and other aerosols, some sectors in the SSP dataset are classified as fossil-fuel related, including aircraft, energy, industrial, shipping, residential/commercial, solvents production and transport, with the other sectors classified as non-fossil related (including agriculture waste burning, agriculture, forest burning, grassland burning, peat burning and waste). Emissions are partitioned into FF and non-FF based on the classification in Hoesly et al. (1). Aerosol emissions are then scaled to radiative forcing based on the present-day forcing values. Radiative forcing of GHGs (CO_2, N_2O and CH_4) are calculated from the corresponding concentrations due to FF and non-FF emissions. Note that the present day forcing is calibrated to reflect our latest understanding of the uncertainty, especially with regard to indirect aerosol-cloud effects of cooling aerosols (ERF_{aci} of –0.7 Wm^{-2} at present), and "all source"

carbonaceous aerosols such as black carbon (+0.6 Wm^{-2} at present for "all sources" versus zero BC, with uncertainty range of 0.25 to 1.0 Wm^{-2}), and co-emitted partially absorbing organic aerosols (–0.5 Wm^{-2} at present-day). The SO_4 (with SO_2 as the precursor) forcing is taken as –0.6 Wm^{-2} at present and NO_x as –0.2 Wm^{-2}.

For the future period analysis, we consider three cases: a weak climate policy baseline case, a middle case with only decarbonization-driven emissions reductions, and an "decarb+targeted" case including policies beyond decarbonization targeting methane, HFCs, and other pollutants. The baseline and "decarb+targeted" scenarios and outputs are selected from the database of Shared Socioeconomic Pathways (SSP)(2). The AIM marker scenario SSP3-7.0, which has only weak climate policy, is selected as the baseline scenario (3). The IMAGE marker scenario SSP1-1.9, which is consistent with the 1.5°C pathway and is the 'cleanest' scenario among the published marker SSP scenarios, is selected as the aggressive climate change mitigation scenario (4, 5). Harmonized outputs for all scenarios are used (6). We use the IMAGE marker SSP1-1.9 scenario as the aggressive climate mitigation scenario for all species, except HFC and N_2O (see below).

We construct the middle "decarbonization" case by partitioning the decrease in emissions in the mitigation case relative to the baseline case into "decarb-driven" and other targeted measures. Most radiatively important species have both fossil fuel and non-fossil fuel related sources Shindell and Smith (7) Extended Data Figure S1 is used as *the present-day* share of current emissions that are FF-related for those species: methane (30%), black carbon (70%), sulfate

(96%), NO_x (96%), and organic carbon (40%). Since this partitioning approach is most valid in the near term, we focus our analysis on the period through 2050.

For future emissions, the FF-related portion of emissions sources is calculated using a scaling factor (akin to emission intensity: a ratio of mass over energy use) to estimate "decarb-driven" emission trajectories based on the decrease in fossil fuel primary energy usage in the mitigation scenario. Under the SSP1-1.9 scenario fossil fuel usage declines rapidly. As a result, FF-related emissions of those species would also decline. Thus, a middle of road pathway ("decarb-driven") is constructed by estimating the reduction in FF-related emission of each species due to decreased use of FF. The residual share is due to non-fossil fuel sources, such as agriculture, waste or use of solid biomass. It is assumed that there is no change in the future in non-FF-related share of the total emissions for the purpose of deriving the "decarb-driven" emissions cut. The CO_2 emissions reduction in this pathway is also associated with carbon capture and sequestration measures.

With the decarb-driven and "decarb+targeted" emission pathway (mostly based on SSP1-1.9), we then convert emissions into atmospheric concentrations for GHG species (CO_2, CH_4, N_2O), and then convert atmospheric concentrations into radiative forcing. Aerosol forcings are scaled based on emission trajectory for the future periods. Tropospheric ozone is not directly emitted, but is produced through chemical reactions involving methane, non-methane volatile organic compounds, and nitrogen oxides. The radiative forcing and warming from tropospheric ozone production are included in the radiative forcing calculated for methane and with the rest scaled with carbon monoxide emission.

Finally, the total and single forcing trajectories, including both historical and future periods, were converted to temperature evolution using an energy balance model RXM (8). The climate equilibrium sensitivity of RXM is taken as 3.05ºC for a doubling of CO_2, very close to the central value of 3ºC (with a *likely* range of 2.5-4ºC) recommended by AR5 and AR6 WGI. The transient climate response in RXM is shown to be 1.8ºC (8), identical to the central value of 1.8ºC (with a *likely* range of 1.4-2.2ºC). RXM has been well tested via comparison with observations, global climate models and models with intermediate complexity (9, 10); see also Figure S3. As shown in Supplementary Figure 21 of ref. (10), RXM yields a higher global mean temperature under SSP1-2.6 toward the end of the 21st century, compared to MAGICC.

Next, we detail the assumption of individual species in order to derive the decarb-driven scenarios.

Methane (CH_4) and tropospheric ozone (O_3). As noted above, tropospheric ozone is not directly emitted, but produced by chemical reactions involving methane, and hence is included as part of the methane emission contribution to warming. Note that the approach used here is simplified in its accounting for the ozone chemical response to methane, the climate response, and the carbon cycle response, leading to differences relative to global composition-climate models, and this may account for a smaller effect than that seen in those more detailed models (11).

The methane emission trend due to decarbonization is constructed by scaling the present-day portion of FF-related emission based on the future reduction of primary energy production. The same emission factor is used for coal and oil/gas because the 1:2 ratio of those present-day energy uses are similar to the Global Methane Budget 2000–2017 finding coal mining contributed 42 Tg CH_4 yr^{-1} and oil and gas contributed 80 Tg CH_4 yr^{-1} (12). Unlike most other fossil fuel emissions, methane comes largely from direct (fugitive) emissions during extraction (on the order of 3.7% of gross gas extracted in the Permian (13)), storage and distribution. Recent satellite observations found that 8 to 12% of global oil and gas methane emissions come from over 1,800 ultra-emitting sources and represent fugitive emissions not included in most current inventories (14). This underestimation from oil and gas would translate to about 2% difference in our approach for partitioning between fossil fuel and non-fossil fuel methane sources since oil and gas methane emissions make up about 22% of total anthropogenic methane emissions (12). Methane is also emitted from abandoned oil and gas wells (15), and often underestimated (16). Current estimates are ~10% of coal-related methane emissions are from abandoned mines (17). Furthermore, halting the use of FF will not reduce methane emissions from abandoned mines or wells. As a result, the actual decarbonization-related reductions are lower than if scaling directly to reduction in FF usage. Thus, in this study, the FF-related methane reduction is scaled down by 0.9 to account for the fact that unlike most other emissions, FF-related methane doesn't come from combustion only but also from extraction, storage, and distribution processes.

Other sources of anthropogenic methane emissions not directly related to fossil fuel include agriculture, animal, and municipal waste (56% of the total anthropogenic emissions) and biomass

and biofuel burning (8%)(12). The non-decarbonization related methane reductions are calculated as the difference between the constructed decarb-related methane reduction and SSP1-1.9.

CFC, HCFC, halon, and HFC. Instead of relying on the SSP database, CFC, HCFC, and HFC forcing are adopted from (18). HFCs are manufactured gases primarily produced for use in refrigeration, air-conditioning, insulating foams, and medical and technical aerosol propellants. HFCs are not co-emitted with fossil fuel combustion. Emissions of HFCs are growing rapidly as HFCs are used to replace ozone depleting chlorofluorocarbons (CFCs), which were previously phased out under the Montreal Protocol, and hydrochlorofluorocarbons (HCFCs), which are now being phased out. HFC emissions in 2016, not including HFC-23, accounted for 0.025 Wm^{-2} of forcing and were projected to increase ten-fold to 0.25 Wm^{-2} by 2050 in the absence of controls agreed under the 2016 Kigali Amendment (19). For the reference scenario, we consider both high and low references as in (18). For the mitigation scenario, we considered KA mitigation, which yields 0.19ºC avoided warming in 2100. A more aggressive scenario with production phaseout in 2020 yields 0.38ºC avoided warming in 2100.

Nitrous oxide (N_2O). Instead of relying on the SSP database, N_2O forcings are adopted from scenarios in (19). In particular, zero anthropogenetic emission after 2020 is adopted as an aggressive illustration of mitigation potential in the future.

For forcing estimates related to aerosol species, we distinguish effective radiative forcing (ERF) due to aerosol-radiation interaction (ERF_{ari}) that is detailed below for individual species and

aerosol-cloud interaction (ERF_{aci}) that is not well constrained and thus difficult to disaggregate among species. Thus, we consider ERF_{aci} separately as a lump-sum “indirect” forcing term associated with the total aerosol emissions, which has an average value of –0.50 Wm^{-2} for 2011 relative to 1850 (uncertainty range of –0.39 to –0.61). We also acknowledge that the ERF_{aci} estimates are subject to the background cloud spatial distribution which could change in future due to feedback process in response to overall global warming. This would impose additional uncertainty to the forcing estimates of future aerosols in this simple climate model framework.

Sulfate/sulfur dioxide (SO_2). The decarb-driven emissions trend for SO_2 is generated by scaling 96% of present-day emissions according to the corresponding future change in primary energy production from coal. Such an approach of scaling to coal use only, rather than the total fossil fuel energy use, is supported by the IPCC Emission Factor Database (https://www.ipcc-nggip.iges.or.jp/EFDB/find_ef.php), in which SO_2 emissions factor for coal are 10-100 times greater than for gasoline and oil. Natural gas combustion only emits a minimal amount of sulfur. The remaining portion of SO_2 emissions reduction (i.e., the difference between constructed pathway and SSP1-1.9), is due to any change in non-fossil fuel related emissions as well as the remainder of the FF-related emissions, which is presumed to be due to other dedicated mitigation measures, such as scrubbing.

Black carbon (BC). Black carbon is not a greenhouse gas, but a powerful climate-warming aerosol that is a component of fine particulate matter (specifically, $PM_{2.5}$) that enters the atmosphere through the incomplete combustion of fossil fuels, as well as biofuels and biomass (20, 21). Taking the ERF_{ari} only and snow albedo effect, BC contributes to an estimated +0.25

Wm^{-2} (22) to +0.9 to 1.1 Wm^{-2} at present (20, 23, 24). In this study, we adopt an average of 0.6 Wm^{-2} based on high and low forcing estimates for "all-source" forcing (0.25 to 1 Wm^{-2}). This gives an average present-day forcing of 0.33 Wm^{-2} for 2011 relative to 1850 (0.13 to 0.53), consistent with AR6 range (0.30±0.2 Wm^{-2} for ERF_{ari} and 0.38 Wm^{-2} including snow albedo effects)(25).

We address entanglement of BC emissions with GHG and other aerosol emissions in two ways: first, we adopt a 70% co-emission factor for BC for FF sources based on (7), such that part of BC mitigation is achieved through decarbonization, while the non-FF related BC, such as from biofuel burning, is included in targeted measures. Second, we include in our discussion of uncertainty that we have not explicitly accounted for the potential enhancement of positive forcing of BC when accounting for the mixing with other reflective aerosol (26). We also note that a portion of the lump-sum ERF_{aci} negative forcing might be attributable to BC, which would lower the total BC forcing (27). Thus, BC forcing has substantial uncertainties in both directions, motivating our use of high and low forcing estimates.

Annual estimations between 4.5 Tg (28) to 7.2 Tg (29) of global black carbon were emitted in 2010 from anthropogenic sources, with forest and savannah fires (not including agricultural waste burning) contributing about 2.3 Tg per year (29). The primary sources of anthropogenic black carbon are combustion of diesel and solid fuels for heating and cooking in households (57%), road and non-road transport (24%), and industry (6%). Other categories include agriculture open field burning (5%), oil and gas flaring (3%), large scale industrial combustion (2%), international shipping and aviation (2%), and waste (1%)(29).

Other cooling aerosols (OC and NO_x). Corresponding portions of other species (OC, NO_x) emissions trends are scaled based on primary energy due to total fossil fuel (coal+gas+oil) using the shares derived from (7). We do not differentiate between fossil fuel sources for OC based on inventories suggesting similar order of magnitude emissions (29).

Land cover and solar activity. Limited forcing due to land cover change and solar activities in the past is factored in, but their potential change in the future is not considered.

References for Supplemental Information

1. R. M. Hoesly, *et al.*, Historical (1750–2014) anthropogenic emissions of reactive gases and aerosols from the Community Emissions Data System (CEDS). *Geosci. Model Dev.* **11**, 369–408 (2018).

2. K. Riahi, *et al.*, The Shared Socioeconomic Pathways and their energy, land use, and greenhouse gas emissions implications: An overview. *Glob. Environ. Change* **42**, 153–168 (2017).

3. S. Fujimori, *et al.*, SSP3: AIM implementation of Shared Socioeconomic Pathways. *Glob. Environ. Change* **42**, 268–283 (2017).

4. D. P. van Vuuren, *et al.*, Energy, land-use and greenhouse gas emissions trajectories under a green growth paradigm. *Glob. Environ. Change* **42**, 237–250 (2017).

5. J. Rogelj, *et al.*, Scenarios towards limiting global mean temperature increase below 1.5 C. *Nat. Clim. Change* **8**, 325–332 (2018).

6. M. J. Gidden, *et al.*, Global emissions pathways under different socioeconomic scenarios for use in CMIP6: a dataset of harmonized emissions trajectories through the end of the century. *Geosci. Model Dev.* **12**, 1443–1475 (2019).

7. D. Shindell, C. J. Smith, Climate and air-quality benefits of a realistic phase-out of fossil fuels. *Nature* **573**, 408–411 (2019).

8. Y. Xu, V. Ramanathan, Well below 2 °C: Mitigation strategies for avoiding dangerous to catastrophic climate changes. *Proc. Natl. Acad. Sci.* **114**, 10315–10323 (2017).

9. J. Chen, H. Cui, Y. Xu, Q. Ge, An Investigation of Parameter Sensitivity of Minimum Complexity Earth Simulator. *Atmosphere* **11**, 95 (2020).

10. R. Hanna, A. Abdulla, Y. Xu, D. G. Victor, Emergency deployment of direct air capture as a response to the climate crisis. *Nat. Commun.* **12**, 368 (2021).

11. United Nations Environment Programme, Climate and Clean Air Coalition, *Global Methane Assessment: Benefits and Costs of Mitigating Methane Emissions* (United Nations Environment Programme, 2021).

12. M. Saunois, *et al.*, The Global Methane Budget 2000–2017. *Earth Syst. Sci. Data* **12**, 1561–1623 (2020).

13. Y. Zhang, *et al.*, Quantifying methane emissions from the largest oil-producing basin in the United States from space. *Sci. Adv.* **6**, eaaz5120 (2020).

14. T. Lauvaux, *et al.*, Global assessment of oil and gas methane ultra-emitters. *Science*, 557–561 (2022).

15. M. Kang, *et al.*, Identification and characterization of high methane-emitting abandoned oil and gas wells. *Proc. Natl. Acad. Sci.* **113**, 13636–13641 (2016).

16. J. P. Williams, A. Regehr, M. Kang, Methane Emissions from Abandoned Oil and Gas Wells in Canada and the United States. *Env. Sci Technol*, 8 (2021).

17. L. Höglund-Isaksson, A. Gómez-Sanabria, Z. Klimont, P. Rafaj, W. Schöpp, Technical potentials and costs for reducing global anthropogenic methane emissions in the 2050 timeframe –results from the GAINS model. *Environ. Res. Commun.* **2**, 025004 (2020).

18. G. J. M. Velders, D. W. Fahey, J. S. Daniel, S. O. Andersen, M. McFarland, Future atmospheric abundances and climate forcings from scenarios of global and regional hydrofluorocarbon (HFC) emissions. *Atmos. Environ.* **123**, 200–209 (2015).

19. World Meteorological Organization (WMO), *Scientific Assessment of Ozone Depletion: 2018.* (2018).

20. T. C. Bond, *et al.*, Bounding the role of black carbon in the climate system: A scientific assessment. *J. Geophys. Res. Atmospheres* **118**, 5380–5552 (2013).

21. Y. Yang, H. Wang, S. J. Smith, P.-L. Ma, P. J. Rasch, Source attribution of black carbon and its direct radiative forcing in China. *Atmospheric Chem. Phys.* **17**, 4319–4336 (2017).

22. G. D. Thornhill, *et al.*, Effective radiative forcing from emissions of reactive gases and aerosols – a multi-model comparison. *Atmospheric Chem. Phys.* **21**, 853–874 (2021).

23. V. Ramanathan, G. Carmichael, Global and regional climate changes due to black carbon. *Nat. Geosci.* **1**, 221–227 (2008).

24. M. Jacobson, Strong radiative heating due to mixing state of black carbon in atmospheric aerosol. *Lett. Nat.*, 695–697 (2001).

25. C. Smith, *et al.*, “Chapter 7: The Earth’s energy budget, climate feedbacks,and climate sensitivity - Supplementary Material” in *Climate Change 2021: The Physical Science Basis. Contribution of Working Group I to the Sixth Assessment Report of the Intergovernmental Panel on Climate Change*, V. Masson-Delmotte, *et al.*, Eds. (2021).

26. H. Matsui, D. S. Hamilton, N. M. Mahowald, Black carbon radiative effects highly sensitive to emitted particle size when resolving mixing-state diversity. *Nat. Commun.* **9**, 3446 (2018).

27. V. Naik, *et al.*, “Chapter 6: Short-lived climate forcers” in *Climate Change 2021: The Physical Science Basis*, Contribution of Working Group I to the Sixth Assessment Report of the Intergovernmental Panel on Climate Change., (Cambridge University Press, 2021).

28. M. Crippa, *et al.*, Gridded emissions of air pollutants for the period 1970–2012 within EDGAR v4.3.2. *Earth Syst. Sci. Data* **10**, 1987–2013 (2018).

29. Z. Klimont, *et al.*, Global anthropogenic emissions of particulate matter including black carbon. *Atmospheric Chem. Phys.* **17**, 8681–8723 (2017).

30. G. Myhre, *et al.*, Anthropogenic and Natural Radiative Forcing. *Clim. Change 2013 Phys. Sci. Basis Contrib. Work. Group Fifth Assess. Rep. Intergov. Panel Clim. Change*, 659–740 (2013).

31. M. Crippa, *et al.*, *Fossil CO2 and GHG emissions of all world countries: 2019 report.* (European Commission. Joint Research Centre Publications Office, 2019).

32. Intergovernmental Panel on Climate Change, "Summary for Policymakers" in *Climate Change 2021: The Physical Science Basis*, Contribution of Working Group I to the Sixth Assessment Report of the Intergovernmental Panel on Climate Change., (Cambridge University Press, 2021).

33. S. Szopa, *et al.*, Summary for Policymakers of the Working Group I Contribution to the IPCC Sixth Assessment Report - data for Figure SPM.2 (v20210809) (2021) https:/doi.org/10.5285/C1EB6DAD1598427F8F9F3EAE346ECE2F (March 12, 2022).

34. J. Fyfe, B. Fox-Kemper, R. Kopp, G. Garner, Summary for Policymakers of the Working Group I Contribution to the IPCC Sixth Assessment Report - data for Figure SPM.8 (v20210809) (2021) https:/doi.org/10.5285/98AF2184E13E4B91893AB72F301790DB (March 12, 2022).

35. P. Forster, *et al.*, "Chapter 7: The Earth's Energy Budget, Climate Feedbacks, and Climate Sensitivity" in *Climate Change 2021: The Physical Science Basis. Contribution of Working Group I to the Sixth Assessment Report of the Intergovernmental Panel on Climate Change*, V. Masson-Delmotte, *et al.*, Eds. (Cambridge University Press, 2021).

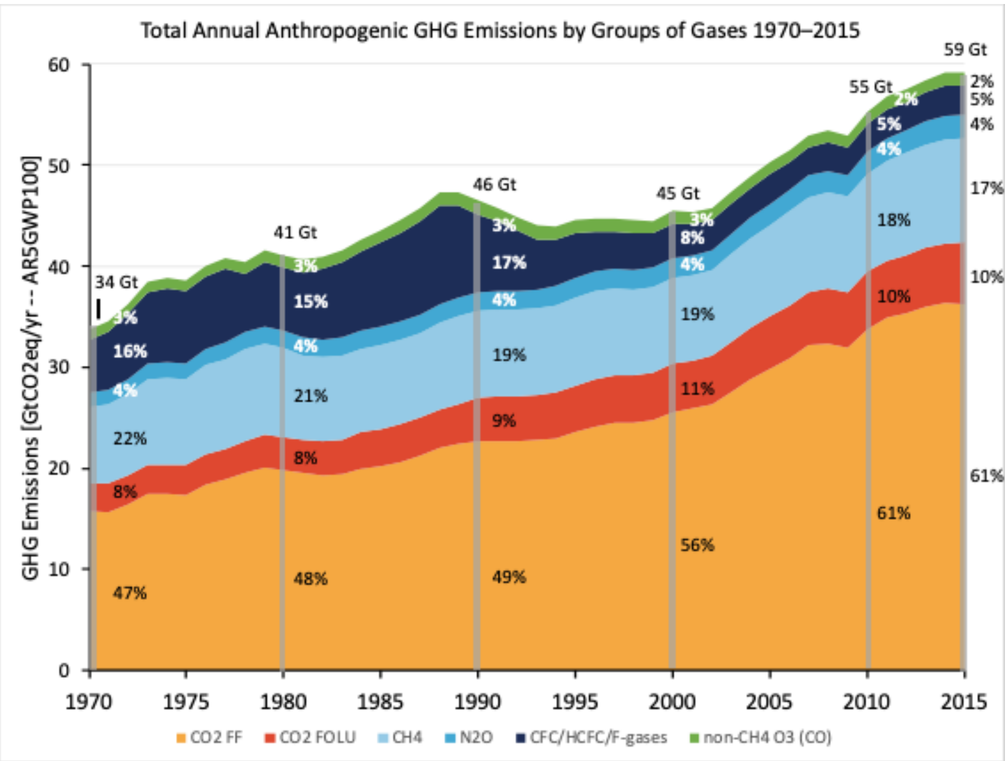

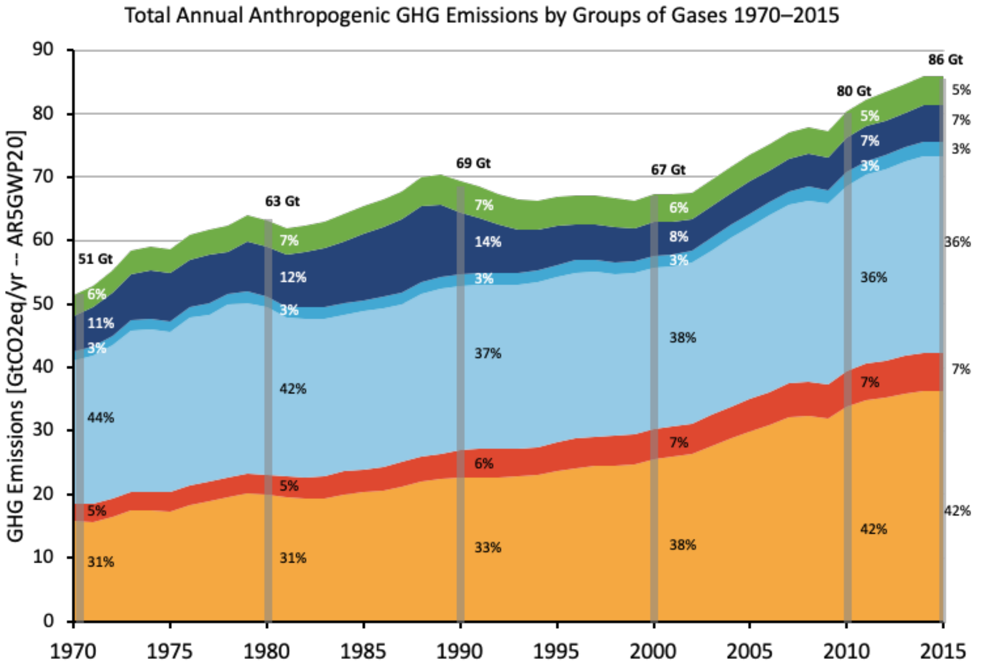

Figure S1. Total GHG emission in $CO_{2\text{-eq}}$. This is revised from IPCC AR5 WGIII figure SPM.1 by taking into account omitted GHGs such as non-CH_4 related tropospheric O_3 (using CO as a proxy for precursors and using GWP_{100} of 2.2 scaled by 0.94 based on Table 8.A.4 in Myhre *et*

al. (30)) from EDGARv5.0 (31), and CFC, HCFC, and halons from NOAA and AGAGE networks. a) using GWP_{100}; b) using GWP_{20} from AR5 (30).

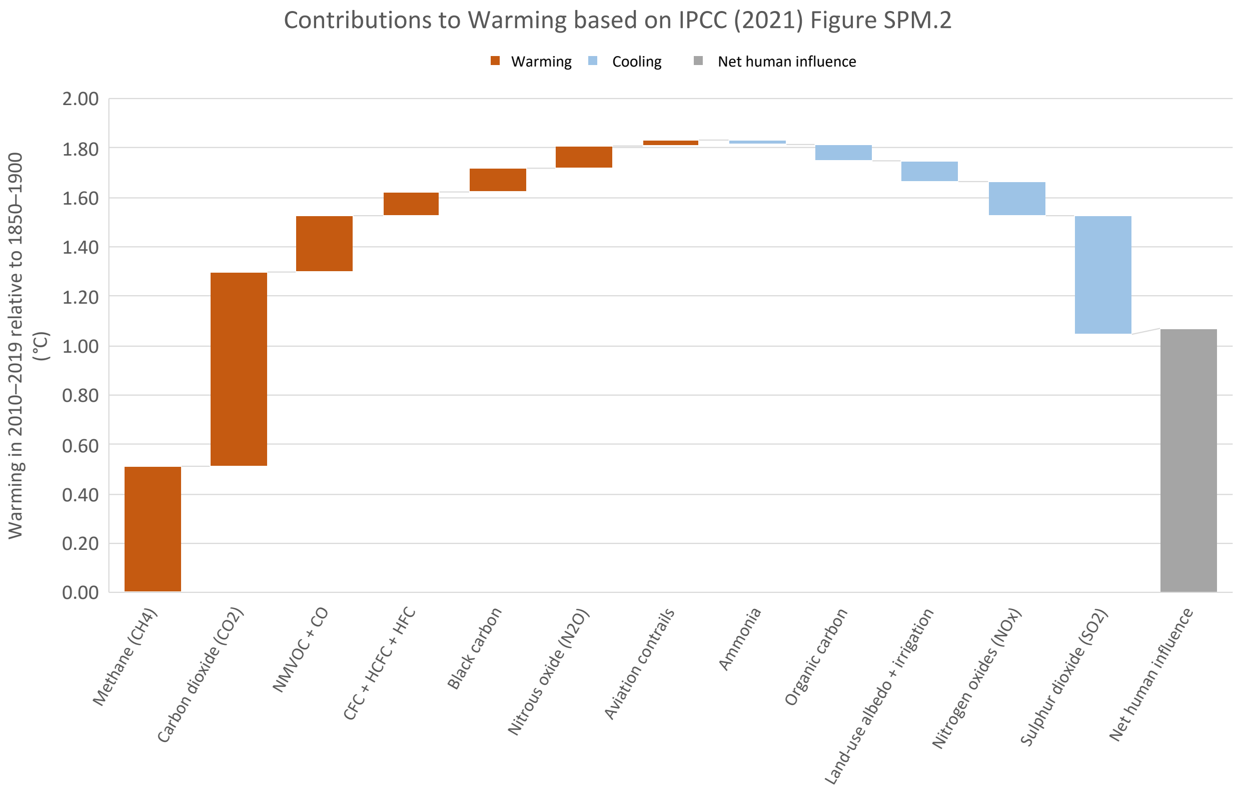

Figure S2. Contributions to observed warming in 2010–2019 relative to 1850–1900 adapted from Figure SPM.2 (32, 33).

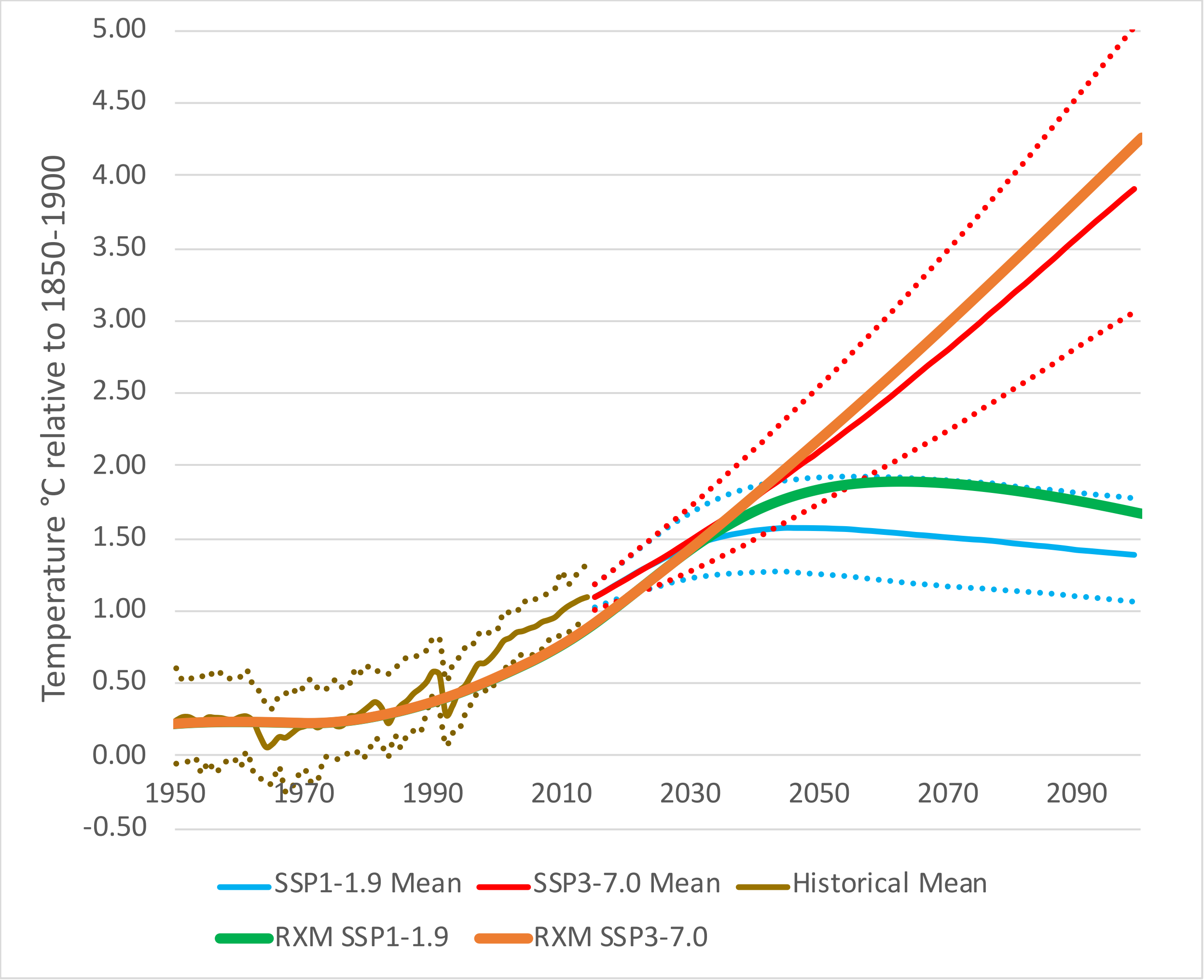

Figure S3. Comparison of RXM model temperature output for SSP1-1.9 (green) and SSP3-7.0 (orange) compared with AR6 WGI Figure SPM8.a for historical (brown), SSP1-1.9 (blue), and SSP3-7.0 (red), wish dashed lines showing 5-95% range (32, 34). RXM reproduces warming and trends for 1950-1990, and gives a slower rate of warming in recent decades (1990-2015) than observed, and higher rate of warming than SSP simulations for 2015-2030. This likely reflects model sensitivity to aerosol forcing. Aerosol forcing increased for 1950-1900 and is projected under SSP1 to decrease for 2015-2030. Figure SPM8.a SSP curves are adjusted by +0.85°C to match observed warming increase between 1850-1990 and 1995-2014.

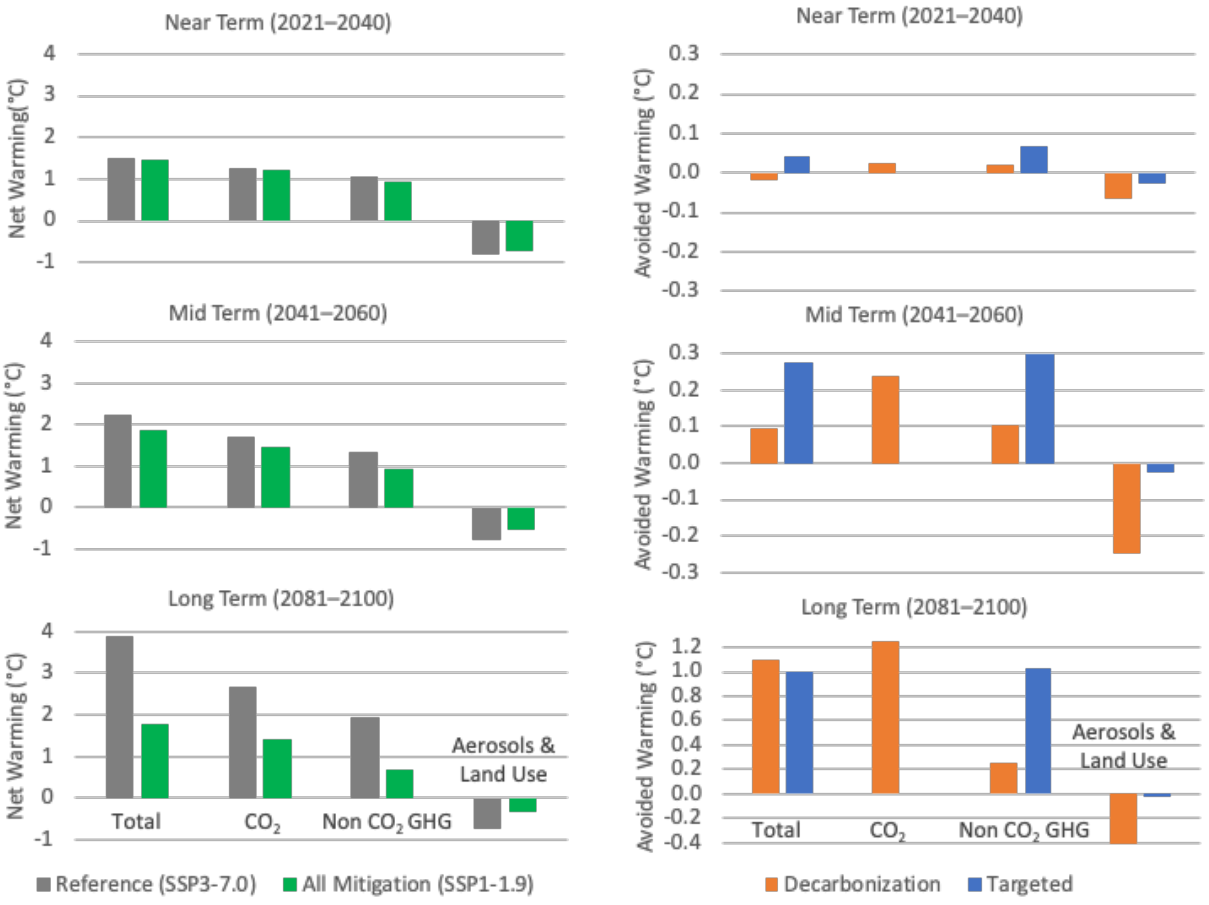

Figure S4. Left panel: net warming (negative means net cooling) relative to 1850 for reference (SSP3-7.0) and "decarb+targeted" mitigation (based on SSP1-1.9) scenarios (2) for all forcers (Total), CO_2, non-CO_2 GHG (includes methane, HFC, ozone, N_2O, CFC+HCFC), and aerosols (warming and cooling) and other (e.g., land use and solar) forcing. Right panel: avoided warming (negative values mean additional warming) relative to the reference scenario partitioned into decarbonization-driven and targeted mitigation contributions (see Table S5).

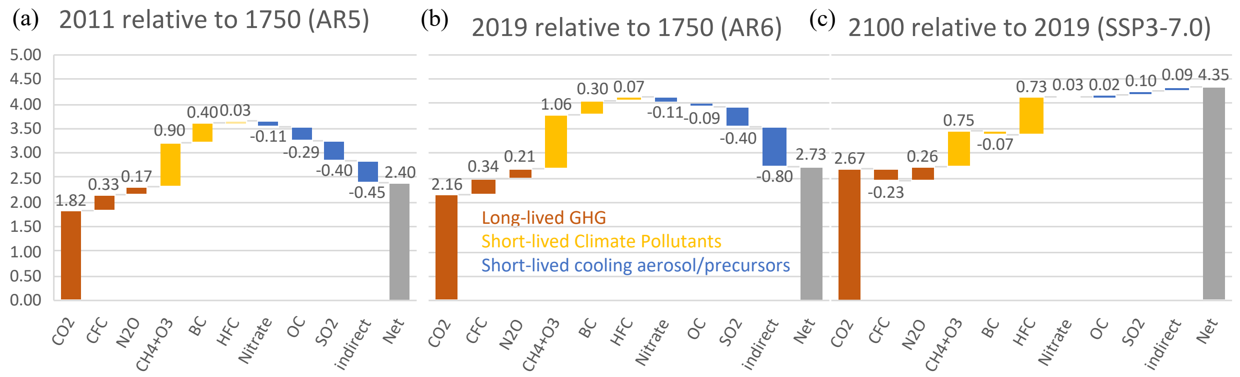

Figure S5. Bar chart version of Figure 1. Positive radiative forcing from long-lived GHG (orange), short-lived GHG, GHG-precursors and black carbon (BC; aerosol-radiation interaction and snow albedo effects only) (yellow), and negative forcing from individual aerosol direct effects (aerosol-radiation interaction) and the total aerosol indirect effects (aerosol-cloud interaction) (blue) in (a) 2011 relative to 1750, from AR5 (30), (b) 2019 relative to 1750, from AR6 (35). (c) The forcing at 2100 relative to 2019, under SSP3-7.0 emissions (2).

Table S1. Present-day radiative forcing of CO_2 and non-CO_2 GHG from various IPCC WGI reports and the climate model used in this study (RXM).

	Forcing (Wm^{-2})		Share of total GHG		Forcing (Wm^{-2})							
Source/ Period	CO_2	Non-CO_2	CO_2	Non-CO_2	LL GHG[a]	SL GHG[b]	CH_4	N_2O	CFC/ HCFC	HFC/ PFC/ SF_6	O_3	Note
RXM 2019–1750	1.85	1.57	54%	46%	2.33	1.09	0.63	0.18	0.30	0.06	0.40	This study using emissions from Hoesly et al. (2018) and SSP3-7.0 (Riahi et al 2017)
RXM 2011–1750	1.62	1.36	54%	46%	2.09	0.89	0.55	0.16	0.31	0.03	0.31	This study using emissions from Hoesly et al. (2018)
AR6 2019–1750	2.16	1.68	56%	44%	2.78	1.1	0.59	0.21	0.38	0.07	0.47	AR6 WGI Table 7.8; Table 7.5
AR5 2011–1750	1.82	1.43	56%	44%	2.32	0.93	0.55	0.17	0.33	0.03	0.35	Sum by concentration; Table 8. SM.6
AR5 2011–1750	1.68	1.57	52%	48%	2.03	1.2	0.97	0.17	0.18	0.03	0.22[c]	Sum by emission; Table 8. SM.6
AR4 2005–1750	1.66	1.35	55%	45%	2.14	0.9	0.55	0.16	0.32	0.017	0.3	AR4 WGI Figure TS.5; AR5 WGI Table 8.6
TAR 1998–1750	1.46	1.19	55%	45%	1.95	0.7	0.5	0.15	0.34	0	0.2	TAR WGI SPM p. 7
SAR 1993–1750	1.57	1.18	57%	43%	n/a	n/a	n/a	n/a	n/a	n/a	0.3	SAR WGI p. 109; AR5 WGI Table 8.6

[a] Long-lived greenhouse gas (LLGHG) includes CO_2, N_2O, CFC/HCFC
[b] Short-lived greenhouse gases (SLGHG) includes CH_4, HFC, tropospheric O_3
[c] Since ozone is not directly emitted, forcing here is taken as the sum of radiative forcing of precursor emissions of CO, NMVOC, NO_x.

Table S2. Historical radiative forcing of GHGs (excluding aerosols) from FF and non-FF sources.

	**FF GHG**	**non-FF GHG**	**FF (CO_2 + non-CO_2) & FOLU CO_2 GHG**	**Non-FF non-CO_2**	**Total GHG**	**Source**
**2011–1750**						**AR5 WGI Table 8.SM.6 sum by emissions partitioned based on Hoesly et al. (2018)**
Radiative forcing (Wm^{-2})	1.65	1.60	2.09	1.16	3.25	
Share of total	51% (38% CO_2; 13% non-CO_2)	49% (13% CO_2; 36% non-CO_2)	64% (51% CO_2; 13% non-CO_2)	36%	100%	
**2011–1750**						
Radiative forcing (Wm^{-2})	1.53	1.45	1.95	1.03	2.97	This study using Hoesly et al. (2018)
Share of total	51% (40% CO_2; 11% non-CO_2)	49% (14% CO_2; 35% non-CO_2)	65% (44% CO_2; 11% non-CO_2)	35%	100%	
**2019–1750**						AR6 WGI Table 7.5 and Table 7.8 (concentration-based) partitioned based on Hoesly et al. (2018) and Shindell and Smith (2019)
Radiative forcing (Wm^{-2})	2.07	1.81	2.58	1.30	3.88	
Share of total	53% (43% CO_2; 11% non-CO_2)	47% (13% CO_2; 34% non-CO_2)	66% (56% CO_2; 11% non-CO_2)	34%	100%	
**2019–1750**						This study using Hoesly et al. (2018) and Shindell and Smith (2019)
Radiative forcing (Wm^{-2})	1.81	1.61	2.24	1.18	3.42	
Share of total	53% (41% CO_2; 12% non-CO_2)	47% (13% CO_2; 34% non-CO_2)	66% (54% CO_2; 12% non-CO_2)	34%	100%	

Table S3. Radiative forcing (including GHG and aerosols) from FF and non-FF sources. Positive forcing from black carbon (BC) for 2011 is taken from IPCC AR5 WGI (0.64 Wm^{-2}) and scaled by the 55% fossil fuel share; the 2019 BC forcing is taken from IPCC AR6 WGI Chapter 7 (0.38 Wm^{-2} including aerosol-radiation interaction and snow albedo effect only) and scaled by 56% fossil fuel share. Negative forcing of cooling aerosol precursors is similarly scaled.

	**FF**	**non-FF**	**FF (CO_2 + non-CO_2) & FOLU CO_2**	**non-FF non-CO_2**	**Total**	**Source**
**2011–1750**						AR5 WGI Table 8.SM.6 sum by emissions partitioned based on Hoesly et al. (2018)
Radiative forcing (Wm^{-2})	1.0 (+2.0; –1.0)	1.6 (+1.9; –0.3)	1.5 (+2.5; –1.0)	1.1 (+1.4; –0.3)	2.6	
Share of total	39%	61%	56%	44%	100%	
**2011–1750**						This study using Hoesly et al., (2018)
Radiative forcing (Wm^{-2})	0.7 (+1.7; –1.0)	1.4 (+1.6; –0.2)	1.1 (+2.1; –1.0)	1.0 (+1.2; –0.2)	2.1	
Share of total	33%	67%	53%	47%	100%	
**2019–1750**						AR6 WGI Table 7.5, 7.8 (concentration-based) partitioned based on Hoesly et al. (2018) and Shindell and Smith (2019)
Radiative forcing (Wm^{-2})	0.9 (+2.3; –1.4)	1.7 (+2.0; –0.3)	1.4 (+2.8; –1.4)	1.2 (+1.5; –0.3)	2.7	
Share of total	34%	66%	54%	46%	100%	
**2019–1750**						This study using Hoesly et al. (2018) and Shindell and Smith (2019)
Radiative forcing (Wm^{-2})	1.0 (+2.1; –1.1)	1.6 (+1.8; –0.2)	1.4 (+2.5; –1.1)	1.2 (+1.4; –0.2)	2.6	
Share of total	39%	61%	55%	45%	100%	

Table S4. Simulated warming in 2015 relative to 1850 (°C) computed based on historical emissions partitioned into fossil fuel (FF) and non-FF sources.

	**Total**	**FF**	**non-FF**
CO_2	0.98	0.68	0.30
CH_4+O_3 + non-CH_4 O_3	0.52	0.19	0.34
BC (direct effects only)	0.37	0.20	0.16
HFC	0.02	-	0.02
CFC	0.17	-	0.17
N_2O	0.10	-	0.10
**Total warming**	2.15	**1.07**	**1.08**
**Total cooling due to all aerosols** (i.e., direct and indirect)	-1.15	**-0.88**	**-0.26**
**Net**	1.01	**0.19**	**0.82**

Table S5. Simulated future warming (avoided warming is shown as negative numbers) partitioned into "decarbonization-related" and "targeted" mitigation measures. These results are calculated using an average value of BC present-day direct forcing at 0.33 Wm^{-2} relative to pre-industrial. For comparison, IPCC AR6 WGI Chapter 6 (27) compares the "all measures" avoided warming for SSP3-7.0 and SSP1-1.9 and finds methane reductions avoid 0.07°C (-0.2 to 0.14°C) in 2040, which is comparable to the 0.09°C of avoided warming found in this study; and similarly for methane, ozone precursors and HFC AR6 finds 0.2°C (0.1 to 0.4°C) in 2040, which is comparable to the 0.18°C found in this study. AR6 finds methane, ozone precursor and HFC reductions of 0.8°C (0.5 to 1.3°C) in 2100, which is comparable to the 1.3°C found in this study, noting that we used the higher estimate for HFC abatement potential here.

Warming (ºC)	2030	2040	2050	2060	2100
Decarbonization					
**CO_2**	**-0.01**	**-0.08**	**-0.22**	**-0.42**	**-1.56**
**SLCP**	**-0.05**	**-0.14**	**-0.25**	**-0.34**	**-0.52**
BC	*-0.03*	*-0.09*	*-0.14*	*-0.19*	*-0.25*
CH4+O3	*-0.01*	*-0.03*	*-0.06*	*-0.09*	*-0.20*
non-CH4 O3	*-0.01*	*-0.03*	*-0.04*	*-0.06*	*-0.07*
Cooling Aerosols	0.09	0.24	0.39	0.51	0.66
**Net**	**0.03**	**0.02**	**-0.07**	**-0.25**	**-1.42**
Targeted measures					
**SLCP**	**-0.09**	**-0.20**	**-0.33**	**-0.48**	**-1.13**
BC	*-0.04*	*-0.07*	*-0.08*	*-0.09*	*-0.11*
CH4+O3	*-0.02*	*-0.06*	*-0.13*	*-0.20*	*-0.52*
non-CH4 O3	*-0.03*	*-0.05*	*-0.06*	*-0.08*	*-0.11*
HFCs	*0.00*	*-0.02*	*-0.06*	*-0.11*	*-0.39*
N_2O	-0.01	-0.02	-0.04	-0.06	-0.18
Cooling Aerosols	0.07	0.10	0.10	0.10	0.14
**Net**	**-0.03**	**-0.12**	**-0.26**	**-0.43**	**-1.17**
All measures					
**CO_2**	**-0.01**	**-0.08**	**-0.22**	**-0.42**	**-1.56**
**SLCP**	**-0.14**	**-0.34**	**-0.57**	**-0.81**	**-1.65**
BC	*-0.07*	*-0.15*	*-0.22*	*-0.28*	*-0.35*
CH4+O3	*-0.02*	*-0.09*	*-0.19*	*-0.30*	*-0.73*
non-CH4 O3	*-0.04*	*-0.07*	*-0.11*	*-0.13*	*-0.18*
HFCs	*0.00*	*-0.02*	*-0.06*	*-0.11*	*-0.39*
N_2O	-0.01	-0.02	-0.04	-0.06	-0.18
Cooling Aerosols	0.16	0.34	0.49	0.61	0.80
**Net**	**0.00**	**-0.10**	**-0.34**	**-0.68**	**-2.59**

Table S6a. Data shown in Figure 1.

**Figure 1(a) 2011 v 1750 (AR5)**				**Figure 1(b) 2019 v 1750 (AR6)**				**Figure 1(c) 2100 v 2019 (This study for SSP3-7.0)**		
	Wm^{-2}	Share total			Wm^{-2}	Share total			Wm^{-2}	Share total
$CH_4 + O_3$	0.90	25%		$CH_4 + O_3$	1.06	26%		Cooling aerosol	0.23	5%
BC	0.40	11%		BC	0.30	7%		$CH_4 + O_3$	0.75	16%
HFC	0.03	1%		HFC	0.07	2%		HFC	0.73	16%
CFC	0.33	9%		CFC	0.34	8%		N_2O	0.26	6%
N_2O	0.17	5%		N_2O	0.21	5%		CO_2	2.67	57%
CO_2	1.82	50%		CO_2	2.16	52%				
**Total warming**	**3.65**			**Total warming**	**4.13**			**Total warming**	**4.64**	
SO_2	-0.40	32%		SO_2	-0.40	29%		CFC	-0.23	77%
OC	-0.29	23%		OC	-0.09	6%		BC	-0.07	23%
Nitrate	-0.11	9%		Nitrate	-0.11	8%				
indirect	-0.45	36%		indirect	-0.80	57%				
**Total cooling**	**-1.25**			**Total cooling**	**-1.40**			**Total cooling**	**-0.29**	
**Net**	**2.40**			**Net**	**2.73**			**Net**	**4.35**	
Source: Table 8.SM.6; BC Table 8.4)				Source: AR6 WGI Table 7.8; Table 7.5; Figure 7.6; BC 7SM-6				Source: RXM for SSP3-7.0 avg BC		

Table S6b. Data shown in Figure 2.

Figure 2(a). Forcing in 2019 relative to 1750						Figure 2(b). Forcing in 2100 relative to 2019					
All CO_2 + non-CO_2 FF (Wm^{-2})			Non-FF non-CO_2 (Wm^{-2})			All CO_2 + non-CO_2 FF (Wm^{-2})			Non-FF non-CO_2 (Wm^{-2})		
CO_2 FF	1.41	57%	$CH_4 + O_3$	0.64	46%	CO_2 FF	2.74	85%	$CH_4 + O_3$	0.49	32%
CO_2 FOLU	0.43	17%	BC	0.20	15%	$CH_4 + O_3$	0.26	8%	HFC	0.73	49%
$CH_4 + O_3$	0.39	16%	HFC	0.06	4%	Cooling aerosol	0.22	7%	N_2O	0.26	17%
BC	0.25	10%	CFC	0.30	22%				Cooling Aerosol	0.02	1%
			N_2O	0.18	13%						
**Total warming**	**2.49**		**Total warming**	**1.38**		**Total warming**	**3.22**		**Total warming**	**1.50**	
**Total cooling**	**-1.06**		**Total cooling**	**-0.22**		**Total cooling**	**-0.12**		**Total cooling**	**-0.25**	
Cooling aerosol	-1.06		Cooling aerosol	-0.22		CO_2 FOLU	-0.07		BC	-0.02	
						BC	-0.05		CFC	-0.23	
**Net**	**1.43**		**Net**	**1.16**		**Net**	**3.10**		**Net**	**1.25**	
